# Nanomaterials for biosensing applications: a review

**DOI:** 10.3389/fchem.2014.00063

**Published:** 2014-08-27

**Authors:** Michael Holzinger, Alan Le Goff, Serge Cosnier

**Affiliations:** Département de Chimie Moléculaire UMR 5250, Biosystèmes Electrochimique and Analytiques, CNRS, University of Grenoble AlpesGrenoble, France

**Keywords:** biosensors, gold nanoparticles, quantum dots, magnetic nanoparticles, nanostructured carbon

## Abstract

A biosensor device is defined by its biological, or bioinspired receptor unit with unique specificities toward corresponding analytes. These analytes are often of biological origin like DNAs of bacteria or viruses, or proteins which are generated from the immune system (antibodies, antigens) of infected or contaminated living organisms. Such analytes can also be simple molecules like glucose or pollutants when a biological receptor unit with particular specificity is available. One of many other challenges in biosensor development is the efficient signal capture of the biological recognition event (transduction). Such transducers translate the interaction of the analyte with the biological element into electrochemical, electrochemiluminescent, magnetic, gravimetric, or optical signals. In order to increase sensitivities and to lower detection limits down to even individual molecules, nanomaterials are promising candidates due to the possibility to immobilize an enhanced quantity of bioreceptor units at reduced volumes and even to act itself as transduction element. Among such nanomaterials, gold nanoparticles, semi-conductor quantum dots, polymer nanoparticles, carbon nanotubes, nanodiamonds, and graphene are intensively studied. Due to the vast evolution of this research field, this review summarizes in a non-exhaustive way the advantages of nanomaterials by focusing on nano-objects which provide further beneficial properties than “just” an enhanced surface area.

## Introduction

As in many different technological sections, nanomaterials have demonstrated their appropriateness for biosensing applications. The intelligent use of such nano-objects led to clearly enhanced performances with increased sensitivities and lowered detection limits of several orders of magnitudes. One general advantage of all nanomaterials is the high specific surface thus already enabling the immobilization of an enhanced amount of bioreceptor units. However, one of the constant challenges is the immobilization strategy used to conjugate intimately the bio-specific entity onto such nanomaterials. Therefore, the technique used to immobilize the enzyme is one of the key factors in developing a reliable biosensor.

Efficient methods for the biofunctionalization of nanomaterials are summarized in reference (Putzbach and Ronkainen, 2013). Briefly, non-covalent approaches representing electrostatic interaction, π–π stacking, entrapment in polymers, or van der Waals forces between the nanomaterial and the biological entity. These principles preserve all specific properties of both, nanomaterial and biomolecule.

Covalent binding: the strategy to attached covalently biomolecules to nanomaterials has an advantage in terms of stability and reproducibility of the surface functionalization and lowers unspecific physisorption. Covalent links can be formed, e.g., by classic amide coupling reactions, cross-linking, or click chemistry. One drawback is the uncontrolled anchoring of the biomolecule which can affect the domain which is responsible for the recognition event.

The immobilization of biomolecules via supramolecular or coordinative interactions: this technique has achieved wide acceptance in recent years in binding biological species to surfaces. The most famous example used in the field of biosensor engineering is the biotin/avidin (or streptavidin) system (Wilchek and Bayer, 1988). Biotinylated biomolecules can be attached to biotinylated substrates via avidin (or streptavidin) bridges. Other affinity systems have been reported like the nitrilotriacetic acid (NTA)/Cu^2+^/histidine complex (Haddour et al., 2005) or the host-guest system adamantane/β-cyclodextrin (Holzinger et al., 2009). The advantage of such systems, compared to the other immobilization methods, is the reversibility, enabling the possibility to regenerate the transducer element. Furthermore, all components like the functionalized transducer surface and the modified bio-receptor can be characterized individually assuring the reproducibility of the constructed biosensor.

According to the chemical composition, almost all nanomaterials can be equipped with appropriate functions via direct functionalization (in some cases already during synthesis), or via coating with functional polymers without affecting their specific properties (Biju, 2014). Such functionalization not only allows the reproducible immobilization of bioreceptor units but can also increase the biocompatibility of these materials.

One particular issue in biosensing devices is that the recognition event is not directly detectable by the used transduction technique. This is the case for affinity biosensors like the immunoreaction between an antigen and its antibody or the hybridization of corresponding DNA strands. Here, further biospecific components (secondary antibodies or DNA strands) modified with labels for optical or electrochemical transduction have to be used. The specific properties of some nanomaterials clearly contributed to the development of “label free” transduction techniques or contribute to clear signal amplifications when used as labels.

## Gold nanoparticles

Within the group of noble metal nanoparticles, gold nanoparticles are mostly used for biosensor application (Li et al., 2010a) due to their biocompatibility, their optical and electronic properties, and their relatively simple production and modification (Biju, 2014).

**Figure 1:**
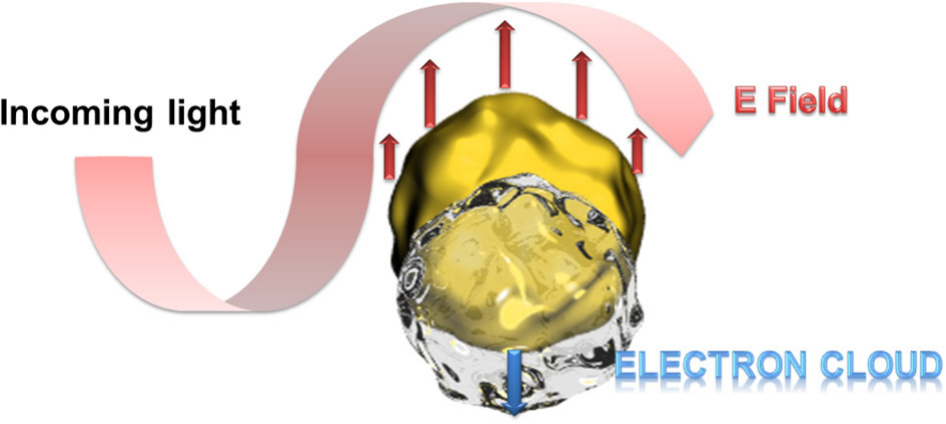
**Schematic presentation of the polarization of the electron density at resonant excitation wavelength**. At particle sizes smaller than the excitation wavelength, the oscillating electrons (surface plasmons) cannot propagate along the gold surface leading to a polarization of the electron cloud at one side of the particle.

Particular interesting is the optical behavior of gold surfaces where irradiation with light of one specific wavelength causes an oscillation of the electrons in the conduction band, called resonant surface plasmons. When the particle size is much smaller than the incident wavelength, the oscillating electrons cannot propagate along the surface as it is for classic surface plasmon resonance (SPR) setups. The electron density is then polarized on one side of the particle where the plasmons oscillate in resonance with the light frequency (Figure 1). This phenomenon was described applying the Mie theory (Mulvaney, 1996; Hao et al., 2004) and is strongly dependent on the size, shape of the nanoparticle and the dielectric constant of its environment (Kelly et al., 2002). This environmental dependency represents a great advantage for (bio)-analytics since the recognition event can result in a change of the oscillation frequency and therefore to a color change of the gold nanoparticles observable with bare eye. In this context, a wide series of efficient colorimetric biosensors were developed for DNA or oligonucleotide detection, or immunosensors (Reynolds et al., 2000; Oldenburg et al., 2002; Liu and Lu, 2004; Xu et al., 2009).

**Figure 2:**
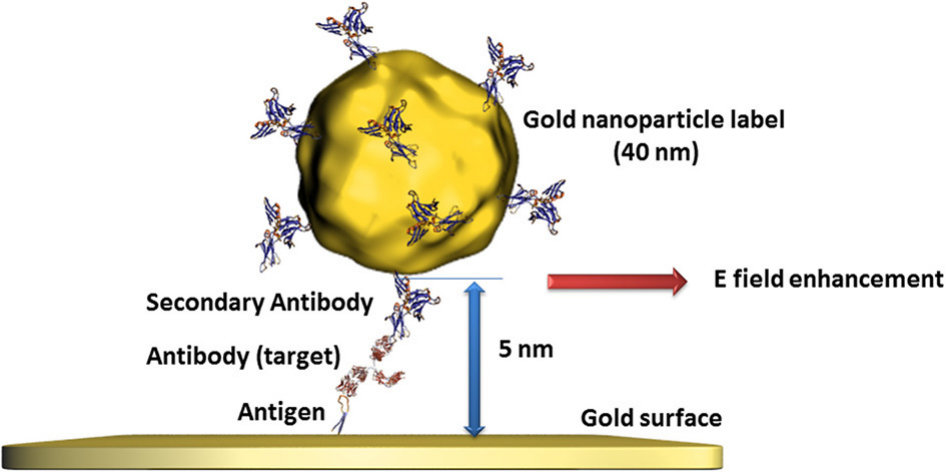
**Illustration of the perturbation of propagating surface plasmons on gold surfaces provoked by gold nanoparticles of defined size and distance leading to an additional change of the evanescent field and therefore to an enhanced signal**.

Gold nanoparticles have also demonstrated their advantages in bioanalysis using SPR transduction. This method is usually based on the change of the dielectric constant of propagating surface plasmons' environment of gold films where the detection of the analyte can be recorded in different ways like the changes of the angle, intensity, or phase of the reflected light (Wijaya et al., 2011; Guo, 2012). Beside the use of a pure gold nanoparticle based SPR transduction replacing the gold film (Pedersen and Duncan, 2005), a clear SPR signal enhancement can be obtained when gold films and gold nanoparticles are used in a sandwich configuration. In fact, the surface plasmons on gold nanoparticles provoke a perturbation of the evanescent field of the gold film in addition to the immobilized bioreceptor unit and the recognized analyte. The optimal configuration of this approach was determined for gold nanoparticles smaller than 40 nm at a distance to the gold film surface of 5 nm (Zeng et al., 2013) as illustrated in Figure 2. In this case, gold nanoparticles serve as labels when attached to secondary antibodies or DNA strands. Even when such labeling needs further preparative steps than label less detection, the signal enhancement is of several orders of magnitudes which is a convincing argument for such an approach.

Gold nanoparticles have also shown their ability to form a powerful transduction platform for single molecule detection. By refractive index sensing of localized surface plasmon resonance (LSPR) coupled with enzyme linked immunosorbent assay (ELISA) using isolated gold nanoparticles of 60 nm sizes. The model enzyme horseradish peroxidase (HRP) was immobilized on these gold nanoparticles via biotin streptavidin linkage. HRP is widely used as label in biosensing applications since it can form colored, fluorescent, or redox active molecules while reducing hydrogen peroxide to water (Veitch, 2004). In the work of Chen et al., HRP oxidized the soluble monomer 3, 3′-Diaminobenzidine (DAB) to insoluble colored polybenzimidazole which aggregates around the enzyme. This aggregation led to an additional shift of the LSPR scattering wavelength enabling the detection of few or even single HRP proteins attached to the gold particles (Chen et al., 2011). The beneficial effects of gold nanoparticles could be validated for high sensitive biosensing using surface enhanced Raman spectroscopy (SERS). Based on surface plasmon assisted signal amplification (Moskovits, 1978) of the vibrational spectrum of adsorbed or immobilized compounds, detection limits also down to the single molecule level could be reached (Nie and Emory, 1997; Hossain et al., 2009; Lim et al., 2011; Saha et al., 2012).

**Figure 3:**
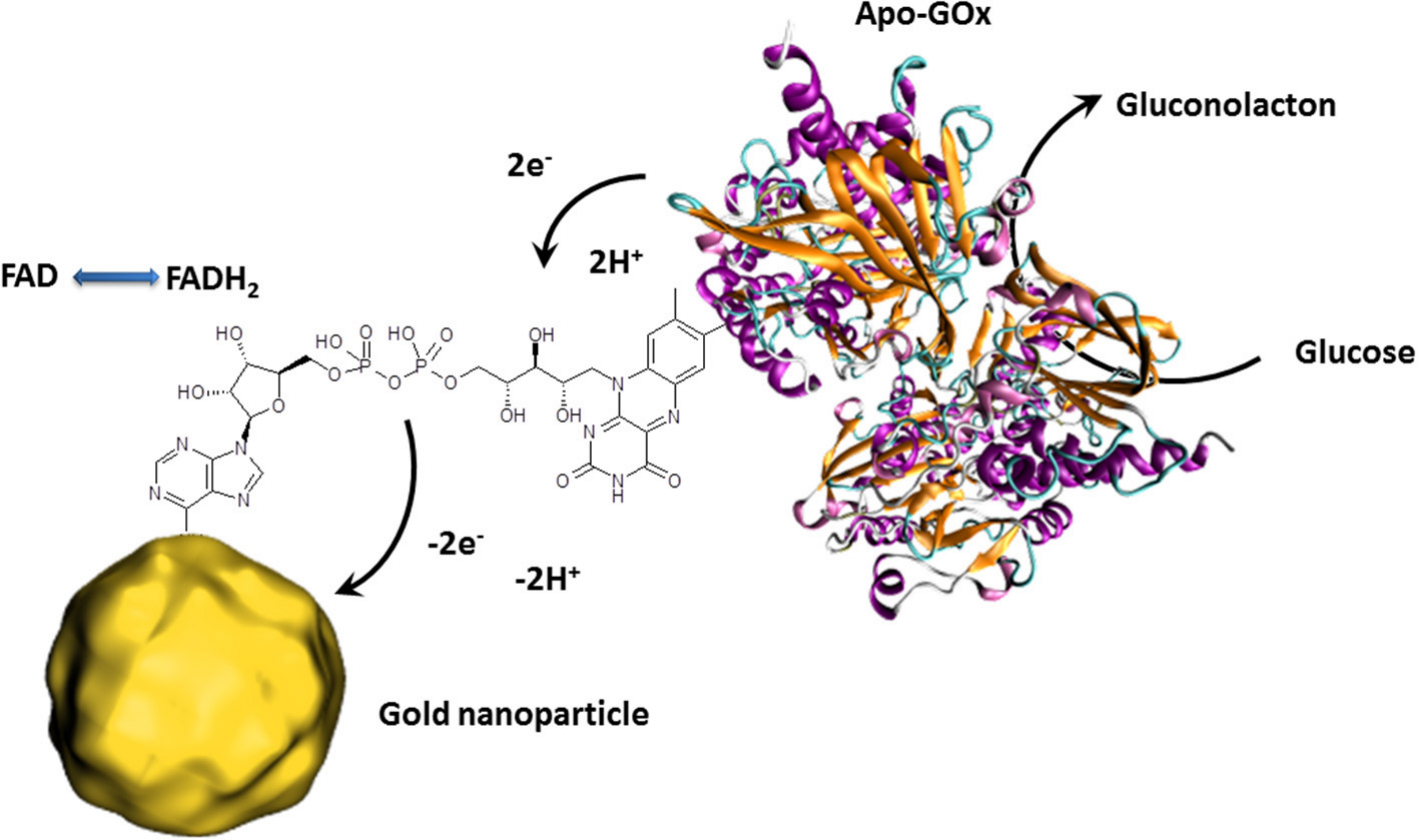
**Schematic presentation of the reconstitution of apo-GOx with FAD modified gold nanoparticles enabling the transfer of electron released by the electrocatalytic oxidation of glucose**. The collected electrons are then directly transferred to the external circuit giving an electrochemical signal.

Besides the outstanding optical properties, gold nanoparticles also have the ability to transfer electrons between a wide range of electroactive biological species and the electrode. This principle is principally used for redox enzyme biosensing where the bioreceptor unit catalyzes the oxidation or reduction of the analyte. In classical electrochemical enzyme biosensors setups, the formed species are oxidized or reduced by the electrode, giving the electrochemical signal. The disadvantage of this approach is that the detectable molecules have to diffuse to the electrode where a non-negligible amount is lost in solution. Gold nanoparticles can act as electron shuttles, i.e., the gold nanoparticles can approach to the redox center of the enzyme regenerating this biocatalyst by transferring the electrons involved in the redox reaction to the electrode. Since such wiring of enzymes prevents the need of enzymatically formed redox species which have to reach the electrode surface, a clear increase of the electrochemical signal is to expect. Particular efficient wiring could be obtained using enzymes where metal ions are involved in the catalytic redox process as it is for HRP (Xu et al., 2006). Nonetheless, the holy grail of enzymes to wire is glucose oxidase (GOx) since its active center is deeply embedded inside the protein structure and contains no metal ion which would facilitate electron transfer (Mena et al., 2005; Willner et al., 2006; Pingarrón et al, 2008). Wiring of GOx is not only a great challenge for glucose sensing applications but also for the design of bioanodes for glucose fuel cells. This is a steady expanding research field since such glucose biofuel cells could generate power to supply implantable medical devices out of body liquids (Cosnier et al., 2014). GOx catalyzes the oxidation of glucose to gluconolactone by releasing 2 electrons. These electrons are generally used to reduce oxygen to hydrogen peroxide. The involved natural electron shuttle is the co-factor flavin adenine dinucleotide (FAD) which is after glucose oxidation in reduced form (FADH_2_). The goal is therefore to provide an electron carrier which regenerates FAD with favored kinetics to avoid the oxygen reduction process. Xiao et al. proposed an original approach by the reconstitution of apo-GOx (GOx without the cofactor FAD) with a FAD modified gold nanoparticle (Figure 3). This work even demonstrated that the as reactivated enzyme showed a 7-fold improved electron transfer turnover rate compared to the natural electron transfer rate to oxygen (Xiao et al., 2003).

These outstanding properties of gold nanoparticles made them promising candidates not only for bioanalytics but also for many other research fields. The particular properties of such gold nanoparticles can be tuned and adjusted. Whatever the desired application, almost any desired shape or size can be obtained using the appropriate synthesis technique. These different morphologies result in different optical, catalytic, and electronic behavior of these gold nanoparticles (Eustis and El-Sayed, 2006).

## Quantum dots

Another prominent example of nanomaterials used for bioanalytics are luminescent semiconducting nanocrystals called quantum dots (QDs). The most studied colloidal QDs are based on cadmium chalcogenides (S, Se, Te) (Murray et al., 1993; Park et al., 2007; Reiss et al., 2009) which provide a very large absorption spectrum with a size-dependent narrow emission spectrum. This phenomenon is due to the varying band gaps of the semiconductor material for different nanocrystal sizes (the bigger the particle the lower the band gap) which leads to distinct emission wavelengths from the recombination of the electron-hole exiton (Weller, 1993). The availability of a large range of emission wavelengths of QDs with different sizes enables efficient multiplexed analysis using classic optical transduction (Geißler et al., 2010; Petryayeva and Algar, 2014). However, structural defects in the crystal lattice can trap the exited electrons or holes leading to non-radiative relaxation (Murphy, 2002). To overcome this issue, core/shell composited with another semiconductor material with a wider band gap range (generally ZnS) were realized to passivate these surface defects and to enhance quantum yields and photo-stability (Dabbousi et al., 1997; Jaiswal et al., 2003). Due to this high photochemical stability of core/shell QDs, this material became a promising alternative to organic fluorophores (Resch-Genger et al., 2008).

In order to provide functional groups for bioreceptor immobilization and also to anticipate possible toxicity issues, QDs are nowadays available with inert or biocompatible coatings (Biju et al., 2010). Thus, almost any kind of biomolecule can be attached to these nanocrystals as long as the photophysical recombination event is not affected.

**Figure 4:**
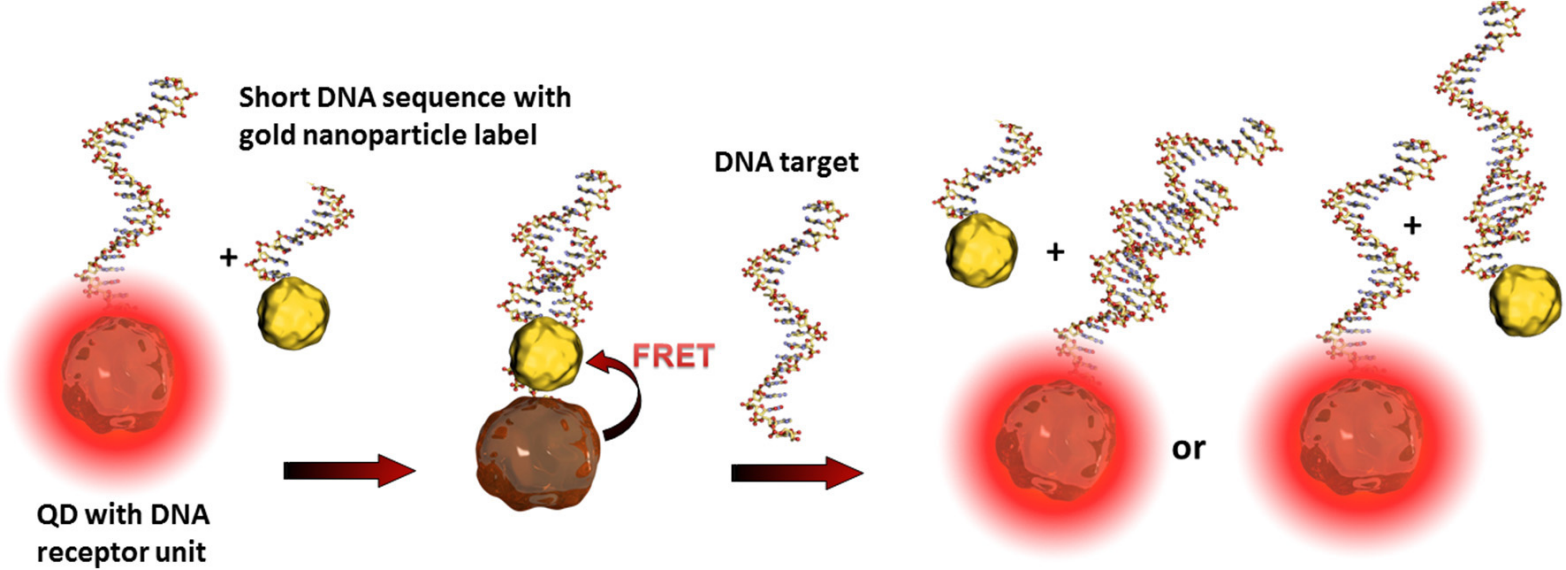
**Illustration of the FRET principle between QDs and gold nanoparticles and the reestablishing of fluorescence after the recognition event**. The target DNA can hybridize either with the receptor DNA, the short sequence of the gold nanoparticle label, or with both. These statistical hybridization events have to be considered for the quantification of the analyte.

Such so-called fluorescence quenching of QDs, described by the Förster (Fluorescence) Resonance Energy Transfer (FRET) (Clapp et al., 2006), is based on a non-radiative energy transfer between the exited QD (donor) and a quencher (acceptor) such as organic fluorophores. Such FRET quenching revealed original approaches used for optical transduction since QD fluorescence reappears when the quencher is removed. This strategy is particularly used for optical DNA and oligonucleotide sensors (Zhang et al., 2005; Freeman et al., 2013). The challenge is to bring the quenching dye in close contact to the QD and to release it after the recognition event. One example deals with the hybridization of a QD tagged receptor DNA and a short corresponding DNA sequence equipped with a gold nanoparticle. As already described, gold nanoparticles are excellent acceptors and are highly efficient QD quenchers. Due to the more favorable hybridization kinetics of the analyte DNA, the short sequence with the gold nanoparticle is released and the QDs' fluorescence reappears where its intensity is correlated to the analyte concentration (Dyadyusha et al., 2005; Dai et al., 2007) as shown in Figure 4.

Another use of non-radiative energy transfer provoking QD fluorescence is called Bioluminescence Resonance Energy Transfer (BRET) (So et al., 2006). Here, a light-emitting protein label transfers the energy to QDs and eliminates the necessity of an external excitation light source. FRET and BRET, together with charge transfer quenching and chemiluminescence resonance energy transfer (CRET) (Huang et al., 2006) are the most common strategies in biosensing applications using QDs as optical transducers (Frasco and Chaniotakis, 2009; Algar et al., 2010; Petryayeva et al., 2013).

**Figure 5:**
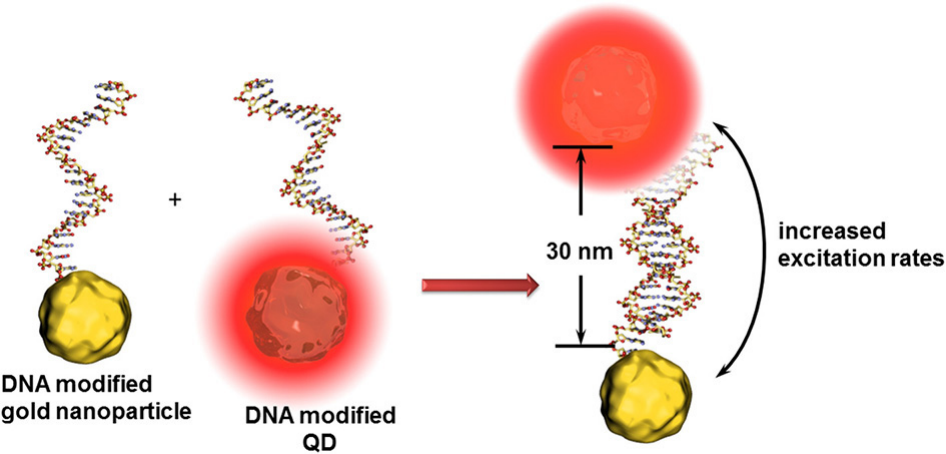
**Principle of the antenna effect of gold nanoparticles enhancing the excitation rates of QDs when positioned at ~30 nm**. This transduction principle might be limited to the identification of analyte DNAs composed of around 15 nucleotides.

For all cases, the distance between the quencher and QD is one of the key factors in the sensitivity of such detection principles. For instance, an enhancement of QD fluorescence was observed when gold nanoparticles are localized at around 30 nm (Figure 5). In this case, the gold nanoparticle acts as antenna (and not as quencher) increasing the excitation rates of the QDs and therefore the fluorescence intensity (Maye et al., 2010). Another effect leading to further example for optical signal enhancement is that QDs can interact with propagating surface plasmons on gold surfaces. These surface plasmons can excite QDs leading to light emission and vice versa - the light induced excited state of QDs are converted to propagating surface plasmons (Wei et al., 2009). Malic et al. demonstrated the beneficial combination of near infrared QDs and gold surfaces for drastic signal enhancement in a SPR imaging biosensor setup (Malic et al., 2011).

QDs have demonstrated their appropriateness in bioanalytics either as transducer unit or as optical labels. A tremendous evolution of QD based biosensors is therefore predictable. Furthermore, the described principles and examples about the use of QDs in bioanalysis let suggest that the combination of different nano-objects and their specific particularity is a promising way for future biosensors with new and original highly efficient transduction techniques.

## Magnetic nanoparticles

Magnetic nanoparticles are promising alternatives to fluorescent labels in biosensor devices. Nanosized magnetic nanoparticles show different magnetic behaviors compared to its bulk material due to the reduced number of magnetic domains (regions of parallel oriented magnetic moments caused by interacting unpaired electrons of an atom) leading to so called superparamagnetic behavior. This means that magnetization can flip the direction randomly within very short time (Neel's relaxation time) and the magnetization appears in average zero in absence of an external magnetic field. This temperature dependent phenomenon disappears by applying an external magnetic field aligning the magnetic moments. Even when this effect seems to be similar to this of classic paramagnetic materials, the magnetic susceptibility of superparamagnets are much higher (Bishop et al., 2009). Such superparamagnetic behavior prevents therefore from attractive or repulsive forces between the magnetic nanoparticles as long as no external magnetic field is applied.

Beside a wide range of ferromagnetic materials, iron oxide is mostly used for bioanalytical applications (Haun et al., 2010). The outstanding advantage using magnetic nanoparticles is the possibility to concentrate the analyte before the detection event. Receptor unit modified magnetic nanoparticles can simply be mixed with the analyte solution and interacts specifically with the specific target. After applying an external magnetic field, the nanoparticles agglomerate and can be separated from the solution. Efficient isolation of DNA strands in complex media was achieved in a fast and efficient manner using silica or gold coated core/shell nanoparticles (He et al., 2007; Li et al., 2011; Min et al., 2014).

**Figure 6:**
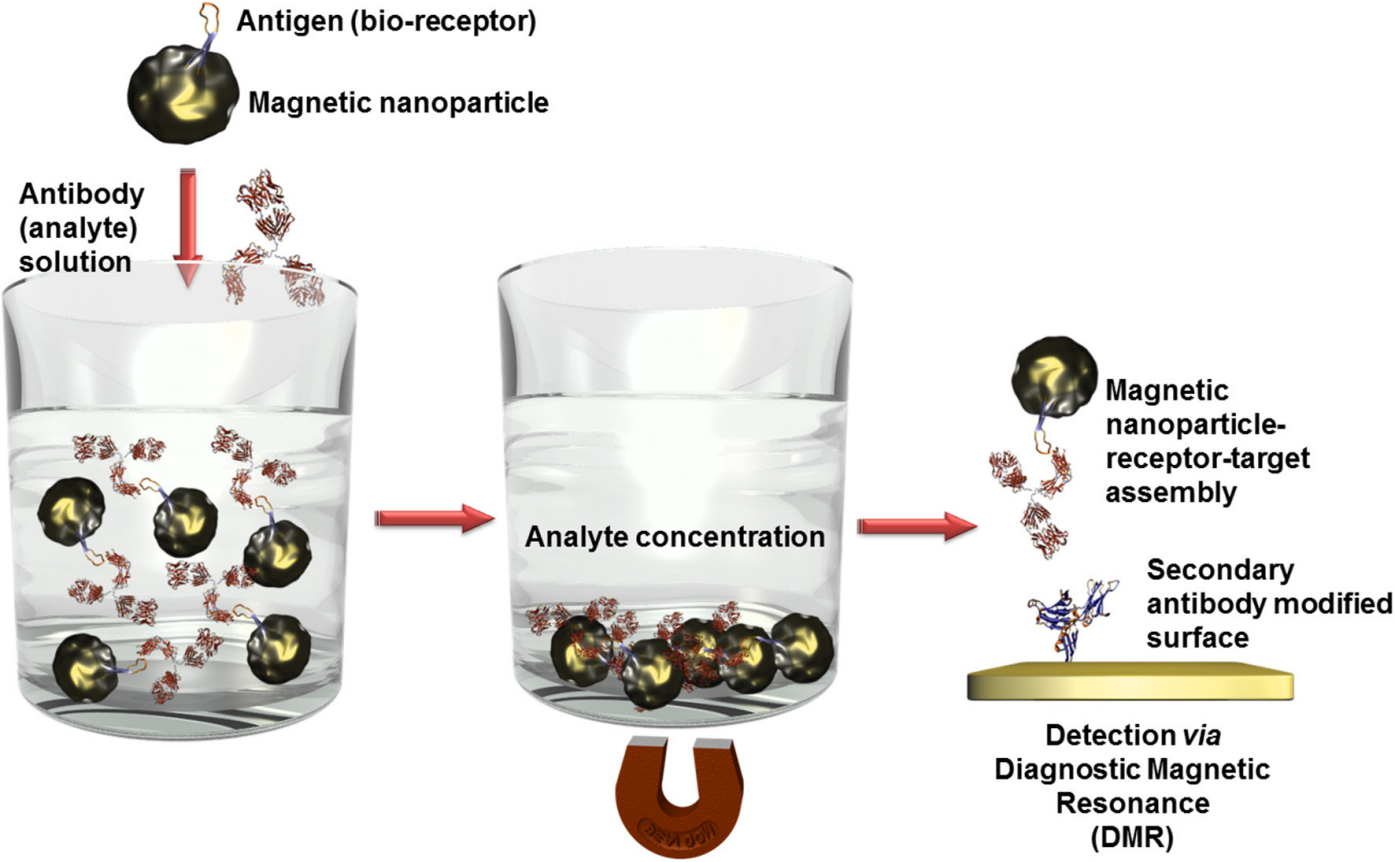
**Scheme of analyte concentration in complex solutions via bioreceptor modified magnetic nanoparticles**. After the recognition event, the captured analytes can be isolated by precipitation of the magnetic nanoparticles in an external magnetic field. When this magnetic field is removed, the nanoparticles can be redispersed. Only the analyte labeled nanoparticles are immobilized on surfaces which are modified with a secondary antibody and thus quantified using diagnostic magnetic resonance transduction.

Besides optical (Bi et al., 2009) or electrochemical (Mejri et al., 2011) detection techniques in combination with other often nanosized labels, magnetic nanoparticles offer a further high sensitive transduction technique in the domain of diagnostic magnetic resonance (Figure 6). In this context, high performant giant magnetoresistance, spin valve, or magnetic tunnel junction biosensors could be developed (Wang and Li, 2008; Konry et al., 2012). Magnetic labels are particular interesting for biosensing applications since biological entities do not show any magnetic behavior or susceptibility and therefore, no interferences or noise is to expect during signal capturing (Tamanaha et al., 2008). For instance, an ultra-high sensitive magnetoresistant biosensor was developed for *Escherichia coli* (Mujika et al., 2009) detection or *Salmonella* were identified in skimmed-milk samples with a limit of detection (LOD) of 1 colony forming-unit (cfu)/mL using a magneto-genosensing setup (Liébana et al., 2009).

A further advantage of magnetic nanoparticles is the possibility to carry the analytes to the transduction platform in microfluidic systems (Konry et al., 2012) enabling even complex multiplexed analyses for the simultaneous detection of different analytes. Nonetheless, these carrier properties are mainly applied for the transport of drugs and genes, or for magnetic resonance imaging (Sun et al., 2008).

## Carbon nanostructures

The beneficial properties of nanostructured carbons such as carbon nanotubes or graphene made them a widely used material as electronic or electrochemical transducer in biosensor devices (Vamvakaki and Chaniotakis, 2007; Valentini et al., 2013). In particular, carbon nanotubes possess the outstanding combination of nanowire morphology, biocompatibility and electronic properties (Battigelli et al., 2013). Therefore, carbon nanotube interfaces present clearly enhanced capacities, e.g., to approach the active sites of a redox enzyme and to wire it to the bulk electrode. Furthermore, their ease and well-documented organic functionalization (Ménard-Moyon et al., 2010) brings new properties to nanostructured electrodes such as specific docking sites for biomolecules or redox mediation of bioelectrochemical reactions. Moreover, CNT films exhibit a high electroactive surface areas due to the natural formation of highly porous three-dimensional networks, suitable for the anchoring of a high amount of bioreceptor units, leading consequently to high sensitivities (Wang, 2005; Le Goff et al., 2011).

Nonetheless, since purely the increase of the specific surface is not discussed here, the presented examples of carbon nanomaterials focus the use of their electronic or electrochemical properties for biosensing applications.

As already mentioned, the nanowire morphology of CNTs enables the approach to the active centers of redox enzyme leading to fast and efficient electron transfers. In few cases, this enzyme wiring spontaneously occurs simply by adsorption of the enzyme on the nanocarbon. The driving force is mostly based on hydrophobic interactions. An important condition is that the hydrophobic domain of the enzyme has to be situated close to its active center for efficient electron transfer after immobilization and orientation.

**Figure 7:**
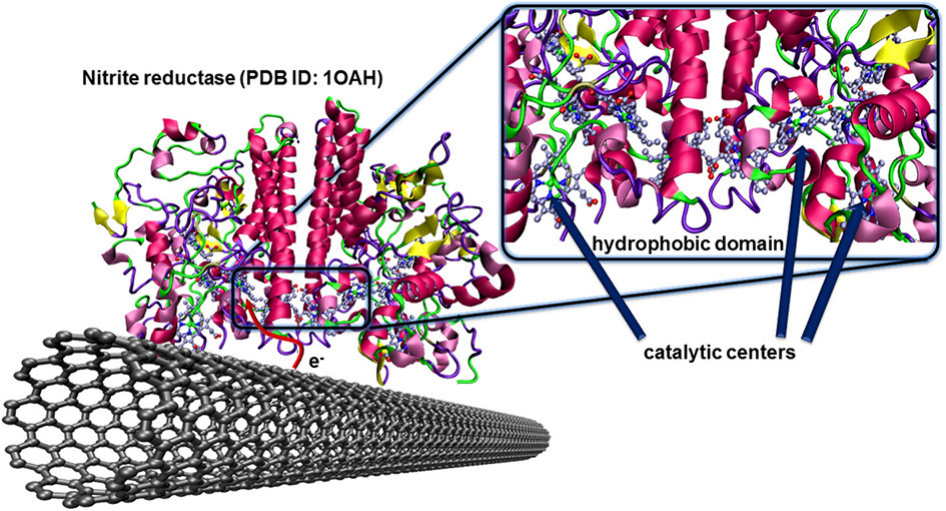
**Oriented immobilization of nitrite reductase from *Desulfovibrio desulfuricans* on CNTs *via* hydrophobic interactions between the enzyme and the carbon nanotube surface**. This naturally occurring adsorption leads to an efficient electron transfer between the enzyme and the conducting surface where the redox center, after catalytic reduction of nitrite ions to ammonia, can directly be regenerated by the electrode.

The nitrite reductase *from Desulfovibrio desulfuricans* is a famous example of such an enzyme having the hydrophobic and catalytic domains at favorable localizations. The multihemic catalytic site for the reduction of nitrite ions is closely placed to a hydrophobic domain of the protein structure as illustrated in Figure 7. After catalytic reduction of nitrite to ammonia, the enzyme, in oxidized state, is directly regenerated by the carbon electrode. This concept was validated for pyrolitic graphite (Silveira et al., 2010a), and CNTs (Silveira et al., 2010b).

Since nature does not always provide such excellent conditions for electron transfers, appropriate functionalization of the carbon material with anchor molecules and/or with redox active species with corresponding redox potentials is necessary to establish so-called mediated electron transfer (via electron shuttle). One particular case of mediated electron transfer is the use of the enzyme specific co-factor. As already demonstrated by Xiao et al. (2003) for glucose sensors using gold nanoparticles as electron carriers, FAD was also attached to carbon nanotubes enabling the wiring glucose oxidase that led to a performant glucose biosensor (Patolsky et al., 2004).

However, the main purpose for efficient enzyme wiring on nanostructured carbon clearly targets bioenergy conversion (Holzinger et al., 2012).

The particular electric properties of CNTs were used in field effect transistor (FET) biosensor setups where changes of the conductivity of the CNT channel or the modulation of the Schottky barrier after the bio-recognition event (Gruner, 2006) led to high sensitivities and low detection limits down to single molecules (Besteman et al., 2003).

**Figure 8:**
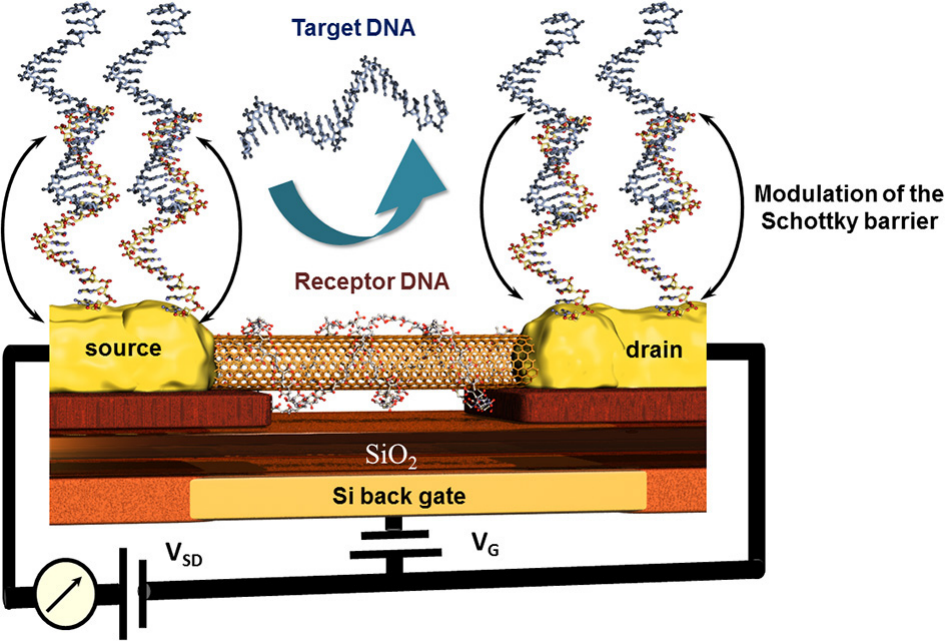
**Principle of the modulation of the Schottky barrier at source and drain electrodes of CNT field effect transistor devices after hybridization of the receptor DNA with the target DNA**. Here, the wrapping properties of DNA around nanotubes inhibit the capability of this receptor DNA to hybridize with its counterpart and thus deactivate indirectly the CNT channel which remains unaffected toward the recognition event.

An original approach for CNT-FET based DNA sensor was proposed by taking advantage of the high affinity of CNTs and DNA strands. The nucleic bases of a ssDNA attach to the CNTs via π −π stacking leading to a wrapping of the DNA around the CNT (Gigliotti et al., 2006) thus eliminating the recognition capacity of this ssDNA toward its counterpart. Tang et al. reported a clear electrical conductance change due to the modulation of energy level alignment between CNT and gold contacts of the CNT-FET device. The receptor unit was attached to the gold electrodes via thioethers where the wrapping of the ssDNA around the conductive CNT channel was accepted. The recognition event was therefore localized only on source and drain, modulating the Schottky barrier and finally the conduction of the CNT channel as sketched in Figure 8 (Tang et al., 2006). This is one of very little examples of CNT based DNA-sensors where the CNTs were not blended in composites to avoid this wrapping effect (Zhou et al., 2009). Else CNT-FET biosensors were mainly developed for enzyme and immunosensing applications (Chao et al., 2005; Gruner, 2006). Some other spectroscopic characteristics of CNTs like NIR photoluminescence and Raman scattering features used for biosensing applications are barely described which is most likely due to the low photoluminescence quantum efficiency (Biju, 2014).

**Figure 9:**
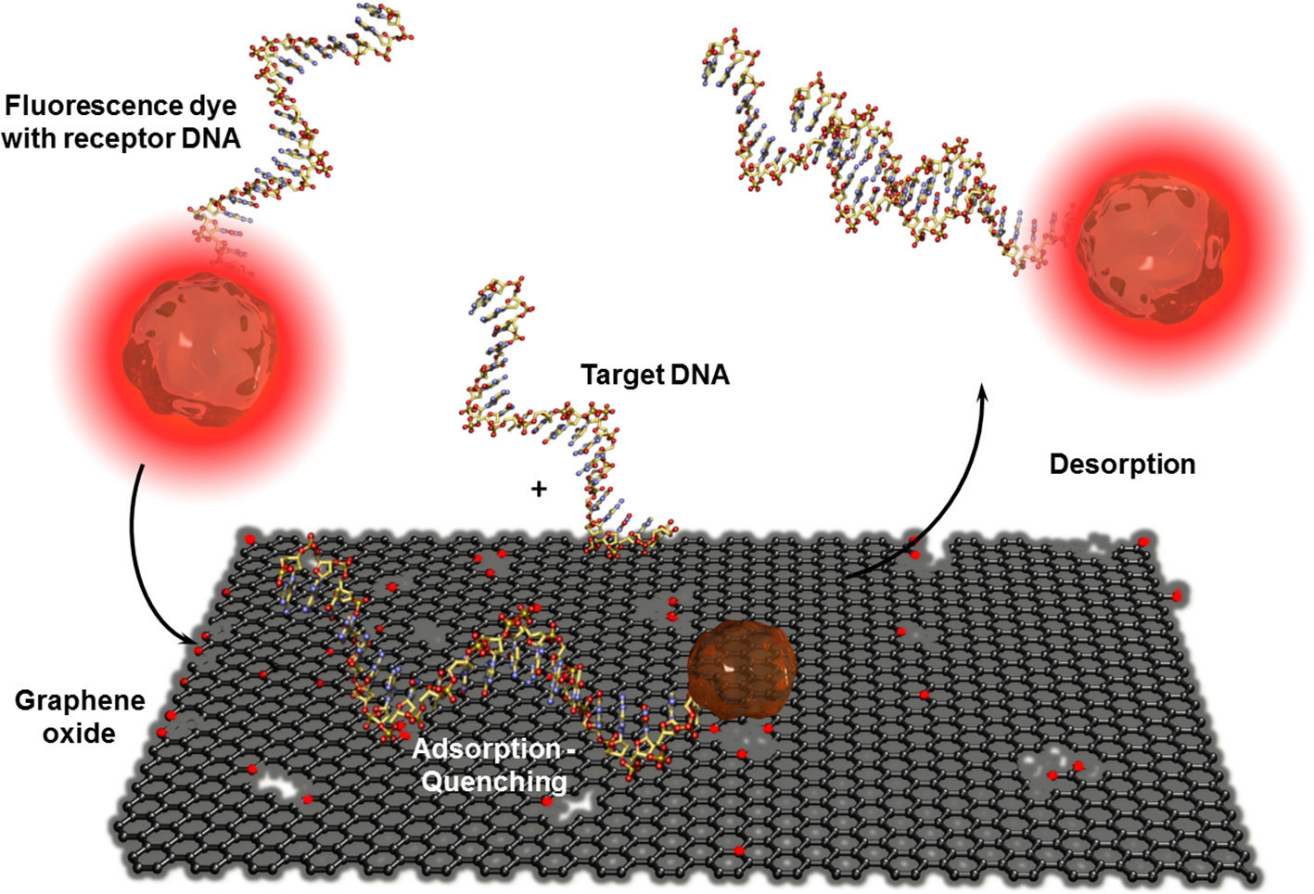
**Adsorption and desorption of a fluorescence dye modified receptor DNA on graphene oxide before and after hybridization with the corresponding target DNA**. Graphene oxide quenches the fluorescence of the dye when the receptor unit is adsorbed. By hybridization with the analyte DNA, the receptor DNA and therefor the dye is released which leads to the reestablishment of the fluorescence.

CNT-based biosensors are clearly more developed than graphene-based biosensors (Yang et al., 2010) but there is a steady increasing interest in this two-dimensional material for bioanalytical applications. Besides the fabrication of isolation of real monolayer graphene sheets via mechanical cleavage using a scotch tape to exfoliate one sheet from highly oriented pyrolytic graphite (HOPG), by CVD on metal foils, or using epitaxy techniques where the graphene layer is formed out of silicon carbide, a wide series of graphite based bulk materials are also called graphene (Bonaccorso et al., 2012). These materials are mostly obtained after mechanic exfoliation (Coleman, 2012; Paton et al., 2014) or chemical oxidation of graphite based on the Hummers and Offeman method (Hummers and Offeman, 1958). This initially called graphitic oxide is now generally known as graphene oxide and allows obtaining soluble carbon oxide sheets of undefined layer composition and sizes. The electric conductivity of this isolating material can be reestablished by chemical, thermal, or electrochemical reduction (Kuila et al., 2013). Even when the exceptional conductivity of real monolayer graphene (Novoselov et al., 2004) cannot be obtained, reduced graphene oxide has other beneficial properties in high performance biosensor devices. As for CNTs, graphene-based materials are mostly used in electrochemical biosensors (Shao et al., 2010; Kuila et al., 2011; Ratinac et al., 2011) or field effect transistor setups (Liu and Guo, 2012) where graphene is principally used as electrode material with enhanced specific surface. Graphene materials can also act as transduction element itself in optical or colorimetric biosensor setups. High efficient graphene-based FRET biosensors were developed in combination with organic dyes (Li et al., 2010b) or quantum dots (Dong et al., 2010) modified receptor units for DNA, aptamer, immuno, or protein sensors (Ma et al., 2012). Contrary to CNTs, ssDNA or oligonucleotide receptors adsorb reversibly to graphene oxide and are released after the recognition event. This allows recovering the fluorescence of the dyes as shown in Figure 9. An interesting example was the adsorption of several ssDNA receptors, each modified with differently colored dyes, leading to a fast multiplex colorimetric DNA sensor (He et al., 2010).

Graphene and CNTs are the most promising nanostructured carbon materials for biosensing applications where each allotrope has its particular advantage as transducer element.

## Conclusion

Nanomaterials became important components in bioanalytical devices since they clearly enhance the performances in terms of sensitivity and detection limits down to single molecules detection. The specific properties of such nano objects also offer alternatives to classic transduction methods. Furthermore, the combination of different nanomaterials, each with its characteristics, to increase even more the performances of biosensors is a well-accepted strategy. Due to the vast number of different nanomaterials all with its own specific properties, only few examples could be mentioned here by emphasizing the principal advantages of such materials. More detailed examples of the use of nanomaterials in biosensor applications are described in the references (Ju et al., 2011; Lei and Ju, 2012), and in the cited review articles.

### Conflict of interest statement

The authors declare that the research was conducted in the absence of any commercial or financial relationships that could be construed as a potential conflict of interest.
